# Nano-Enabled Strategies for the Treatment of Lung Cancer: Potential Bottlenecks and Future Perspectives

**DOI:** 10.3390/biomedicines11020473

**Published:** 2023-02-06

**Authors:** Mohammed Kanan Alshammari, Eman Yaser Almomen, Kholoud Falah Alshahrani, Shroog Farhan Altwalah, Mehnaz Kamal, May Faiz Al-Twallah, Suheir Hassan Alsanad, Mariam Hassan Al-Batti, Faisal Jarallah Al-Rasheed, Abdulaziz Yousef Alsalamah, Mohammed Bader Alhazza, Faisal Abdu Alasmari, Abida, Mohd Imran

**Affiliations:** 1 Department of Clinical Pharmacy, King Fahad Medical City, Riyadh 12211, Saudi Arabia; 2 Saud Al-Babtain Cardiac Center, Nursing Quality, Dammam 32245, Saudi Arabia; 3 College of Pharmacy, King Khalid University, Abha 62463, Saudi Arabia; 4 College of Pharmacy, University of Hail, Hail 55424, Saudi Arabia; 5 Department of Pharmaceutical Chemistry, College of Pharmacy, Prince Sattam Bin Abdulaziz University, Al-Kharj 11942, Saudi Arabia; 6 Department of Pharmaceutical Care, Northern Area Armed Forces Hospital, Hafar Albaten 10018, Saudi Arabia; 7 Department of Pharmacy, Maternity and Children Hospital, Dammam 63430, Saudi Arabia; 8 Pharmaceutical Care Department, Ministry of National Guard Health Affairs, Qassim 51911, Saudi Arabia; 9 Pharmaceutical Care Department, Ministry of Defense Health Services, Qassim 51911, Saudi Arabia; 10 John Hopkins Armco Healthcare, Dhahran 34465, Saudi Arabia; 11 Inpatient Pharmacy Department, National Care Hospital, Riyadh 11541, Saudi Arabia; 12 Department of Pharmaceutical Chemistry, Faculty of Pharmacy, Northern Border University, Rafha 91911, Saudi Arabia

**Keywords:** nanotechnology, lung cancer, passive targeting, drug resistance, active targeting, clinical studies, patent

## Abstract

On a global scale, lung cancer is acknowledged to be the major driver of cancer death attributable to treatment challenges and poor prognosis. Classical cancer treatment regimens, such as chemotherapy or radiotherapy, can be used to treat lung cancer, but the appended adverse effects limit them. Because of the numerous side effects associated with these treatment modalities, it is crucial to strive to develop novel and better strategies for managing lung cancer. Attributes such as enhanced bioavailability, better in vivo stability, intestinal absorption pattern, solubility, prolonged and targeted distribution, and the superior therapeutic effectiveness of numerous anticancer drugs have all been boosted with the emergence of nano-based therapeutic systems. Lipid-based polymeric and inorganic nano-formulations are now being explored for the targeted delivery of chemotherapeutics for lung cancer treatment. Nano-based approaches are pioneering the route for primary and metastatic lung cancer diagnosis and treatment. The implementation and development of innovative nanocarriers for drug administration, particularly for developing cancer therapies, is an intriguing and challenging task in the scientific domain. The current article provides an overview of the delivery methods, such as passive and active targeting for chemotherapeutics to treat lung cancer. Combinatorial drug therapy and techniques to overcome drug resistance in lung cancer cells, as potential ways to increase treatment effectiveness, are also discussed. In addition, the clinical studies of the potential therapies at different stages and the associated challenges are also presented. A summary of patent literature has also been included to keep readers aware of the new and innovative nanotechnology-based ways to treat lung cancer.

## 1. Introduction

Lung cancer has significantly increased the disease burden globally. The high lethality of lung cancer is attributable to the lack of early diagnostic techniques because more than 50% of patients are diagnosed at a later stage of the malignancy (stage IV) and have a poor prognosis for survival [1]. The complexity of the treatment process is further increased by the lack of accessibility of the deeper lung regions when treated by conventional therapy [2].

**Figure 1:**
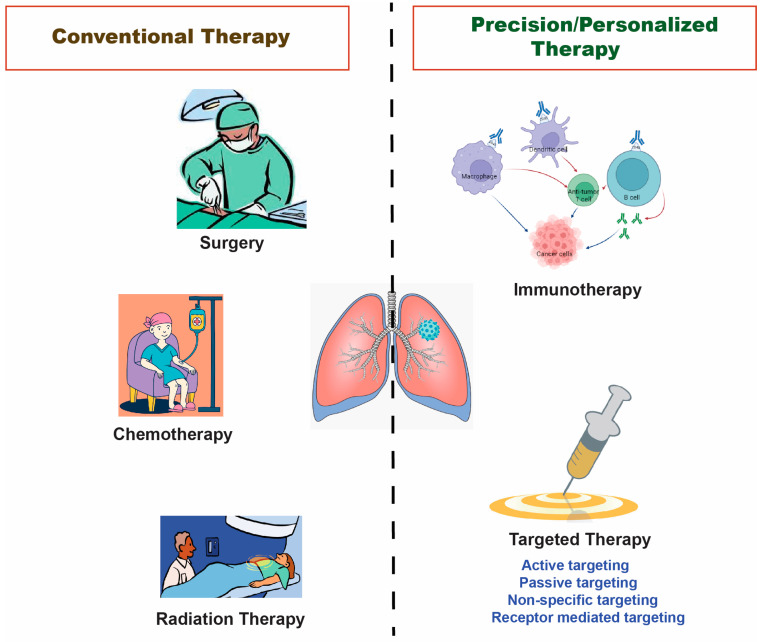
The figure illustrates the various ways that are now being taken to treat lung cancer.

Surgery in the later stages of the malignancy is the only treatment option for non-small-cell lung cancer (NSCLC). The prognosis for locally advanced, incurable non-small cell lung cancer is being gradually improved by the inclusion of chemotherapy to radical radiotherapy and new radiotherapy modalities. Chemotherapy offers individuals with non-small-cell lung cancer a moderate increase in survival [3]. Conventional cancer chemotherapy has serious drawbacks in the form of severe side effects and nonspecific anticancer medication delivery [4,5]. In addition, immunotherapy represents one of the most important and promising therapies employed for lung cancer treatment. Since lung cancer is a solid tumor with a low antigenicity and a heterogenic character, it escapes the host’s immune system. In the tumor environment, a complicated process inhibits the cytotoxic anticancer impact. This suppression of the immune response is greatly aided by the presence of regulatory T cells (Tregs). Foxp3 was highly expressed in lung cancer cells and tumor-invading lymphocytes (TIL). The CTLA4 protein, which inhibits T cell activation, is ubiquitously expressed on Tregs. Patients with lung cancer exhibited lymphocytes with higher CTLA4 expression. In addition, numerous cytokines that can have a suppressive immunological effect in the cancer microenvironment are released by both immune and cancer cells. Transforming growth factor (TGF) and IL-10 are noted to be the most active among them. These are the major factors that lead to immune system dysregulation in lung cancer development [6]. The therapeutic interventions aimed at restoring the correct immune function include chimeric antigen receptor (CAR) T cell immunotherapy [7], combining local therapy with chimeric antigen receptor (CAR) T cell immunotherapy which may control the tumor microenvironment (TME) and increase the killing capability of CAR T cells in the said tumor population [8], targeted therapies including targeting epidermal growth factor receptor (EGFR), anaplastic lymphoma kinase (ALK), c-ros oncogene 1 (ROS-1), Neurotrophic tropomyosin receptor kinase (NTRK), mesenchymal-epithelial transition (MET) factor receptor, rearranged during transfection (RET) genes, and immunotherapeutics which included immune checkpoint inhibitors, cancer vaccines, and adoptive T cell therapy [9]. Although the mainstays of cancer treatment for many decades have been surgery, chemotherapy, and radiation, recent developments are enabling healthcare providers to individualize their patients’ care with precision medicine. The current approaches to lung cancer treatment are depicted in Figure 1.

The clinical utility of the traditional therapeutic approaches encounters numerous limitations, such as serum breakdown, rapid clearance from the biological system, immune response aggravation, off-target side effects, and subpar cellular penetration, which often leads to treatment failure [10].

**Figure 2:**
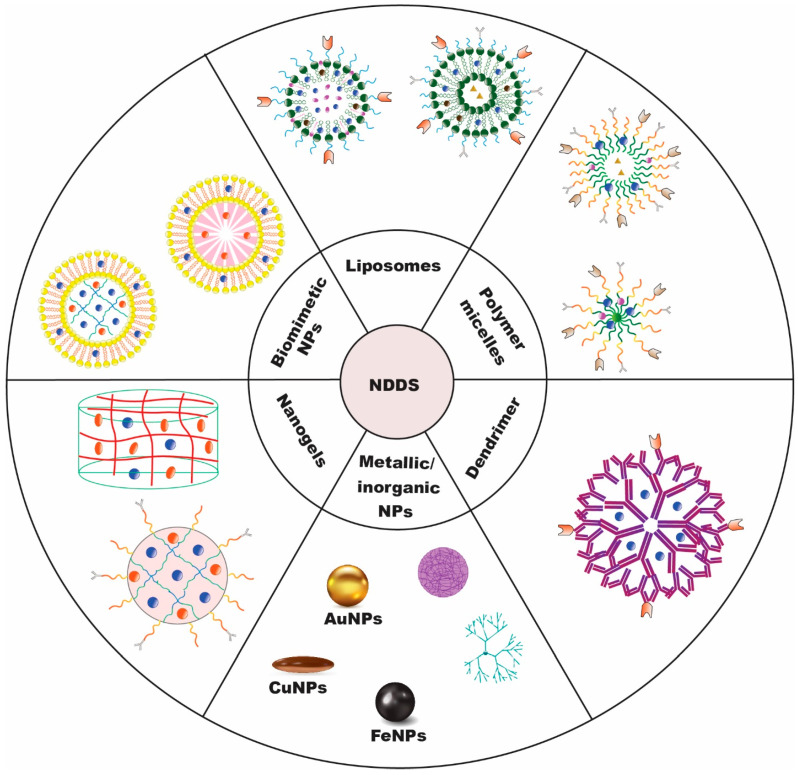
Nanocarriers for delivery of chemotherapeutics to solid tumors [22].

In view of the challenges mentioned earlier, a novel strategy, such as the application of nanoscale materials or the advent of nanotechnology, is needed [11]. The application of nanotechnology has garnered attention owing to its usage in varied therapeutic domains [12,13,14]. Several of these nanoscale compositions have shown positive outcomes. As a result, many nanocarriers are employed to deliver chemotherapeutics to solid tumors (Figure 2). Nano-enabled lung cancer treatment and/or diagnostic systems can efficiently cross the bronchial epithelial barrier and accumulate in deep lung areas due to their nanoscale nature. Many nanoscale delivery systems are being researched to treat an array of malignancies, including lung cancer [15,16,17,18,19,20,21].

## 2. Targeting of Nano-Formulations for Lung Cancer

**Figure 3:**
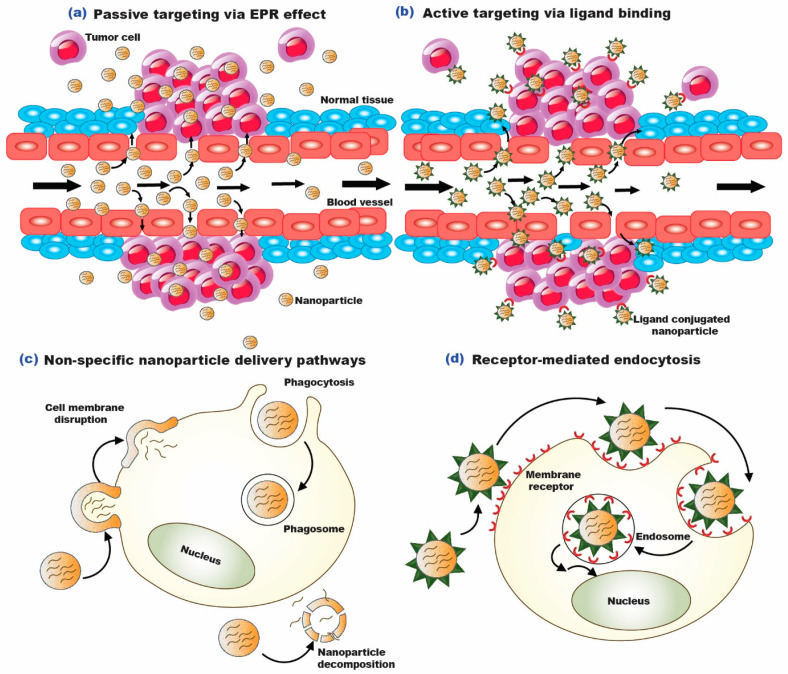
Targeting apoptotic pathway: (**a**). Passive targeting via EPR effect; (**b**). Active targeting via ligand binding; (**c**). Nonspecific nanoparticle delivery pathways; (**d**). Receptor-mediated endocytosis [28].

Long-circulating nanosized therapeutics are kept in the tumor bed by diminished lymphatic outflow after preferentially penetrating tumor tissue through the permeable tumor vasculature. The said phenomenon is termed the enhanced permeability and retention (EPR) effect [23]. Due to the unique traits of the tumor milieu, which are not typically encountered in normal healthy tissues, passive targeting makes it easier for nano-vectors to be deposited at the tumor site [13]. Along with variables intrinsic to the nanoparticle, such as particle size, shape, and zeta potential, the tumor microvasculature also performs a function in the delivery of nanoparticles [24]. Targeting technologies have advanced significantly over the years to improve the preferential internalization of nanoparticles into tumor cells [25]. Targeting markers overexpressed by cancerous cells require the attachment of biorecognition molecules or ligands to the surface of the formulated nano-vectors. These tactics have been given the moniker “active targeting”, demonstrating greater specificity and effectiveness in attaining the desired consequence [26]. Some of the widely used targeting ligands are transferrin, folic acid, hyaluronic acid, aptamers, etc. [27]. The pictorial representation of passive and active targeting strategies is presented in Figure 3.

The FDA has approved a handful of nano-formulations for use in the treatment of cancer, including stealth TMPEGylated liposomal doxorubicin (Doxil or Caelyx). In 1995, the FDA approved Doxil^®^ as the first nanoparticle-based drug delivery system. In phase I/II research, patients with SCLC and NSCLC showed significant responses to Doxil when combined with other standard therapies and supportive growth factors [29]. Albumin-bound paclitaxel, commonly known as Abraxane, was another FDA-approved nano-formulation for metastatic NSCLC. Either alone or in combination with other chemotherapeutics, Abraxane has demonstrated superior therapeutic efficacy with reduced toxic effects [30].

The possible application of nanotools entrapping chemotherapeutics for lung cancer treatment is elaborated throughout this review. Possible strategies to boost therapeutic efficacy, such as combinatorial drug therapy and modalities to reverse drug resistance in lung cancer cells, are elaborately discussed. While many of these approaches are still in clinical phases that are recruiting participants, some of them have already been assessed in numerous clinical trials and are awaiting results or have recently been published; results are also discussed in a sub-section of this article.

### 2.1. Passive Targeting of Nano-Formulations for Lung Cancer

A surge of NPs enters tumor locations because of the atypical blood vessel development and absence of typical vascular basement membrane features. The “passive targeting” method of nanoparticle buildup in tumors is made possible by a phenomenon known as the EPR effect. Drug accumulation occurs at the tumor site due to this mechanism, which is predicated on the physiochemical characteristics of nanoparticles and intrinsic tumor traits [31]. Through the EPR effect, NP features such as size, shape, and surface qualities influence the effectiveness of drug delivery. It is preferable for nanoparticles between 40 and 400 nm to stay in the biological environment for a protracted time. The risk of NPs being cleared by the spleen and kidney depends on the type of NPs formulated. Hence, it is critical to formulate NPs with adequate physicochemical attributes to prevent the elimination of NPs from systemic circulation [32,33,34]. Compared to other cancer medication formulations, liposome formulations for managing non-cell lung carcinoma are quite constrained. Boulikas and co-workers designed lipoplatin, which included cisplatin in liposomes, and the formulation significantly lessened nephrotoxicity in rats [35]. Lipoplatin, which had positive results from clinical testing, may be a treatment option for lung cancer in the coming years.

The advancement of novel methods and approaches to combat malignancy has been greatly aided by the proposal of gene therapy to combat cancer development, but the effectiveness of the proposed strategies has not yet reached the level needed to fully realize the potential of gene therapy in the clinic. Despite the wide range of gene modulation techniques available, such as gene silencing, antisense treatment, RNA interference, and gene and genome editing, it has proven difficult to effectively transport these effectors to the targeted cell or tissue. A number of cutting-edge platforms have been proposed by nanomedicine to avoid this problem [36]. In the past decades, the synthesis of nanoparticles for the transport of DNA or pDNA has received much attention in cancer therapy. Owing to their superior gene transfection and silencing effectiveness, recent research implies that the targeted administration of short RNAs such as siRNA and miRNA is growing in popularity in cancer therapy [37]. Employing mice carrying aggressive adenocarcinoma cells, a team of researchers has proven the use of jetPEITM in combination with cyclin-B1 and survivin sticky siRNA (ssiRNA) to maximize systemic distribution for lung tumor metastasis [38]. The metastasis inhibition of lung tumors was demonstrated to be 64.0 ± 17.0% and 67.0 ± 7.5% upon treatment with cyclin-B1 and survivin ssiRNA/PEI complexes. The PEI/ssiRNA complex’s particle sizes were noted to be 40 to 70 nm with a zeta potential close to +35 mv. Nevertheless, despite the fact that the LD_50_ was observed to be 25-fold higher than the IC_50_ values, the cytotoxicity of this PEI/ssiRNA complex could not be ignored. A chemotherapeutic drug regimen has been used with nanoparticle-based gene therapy to increase treatment effectiveness. For the in vitro trials of treating NSCLC (A549), researchers created a gene delivery system that combined the controlled release of the chosen chemotherapeutics [39]. The signal transducer and activator of transcription-3 (Stat-3) siRNA was deposited on the surface of the paclitaxel-entrapped PLGA nanoparticles by the PEI coating via electrostatic interactions. The results demonstrate that the combinatorial formulation inhibited Stat-3 expression and increased cellular death by accumulating paclitaxel in A549 cells. Topotecan (TPT) has limited clinical use owing to related pharmacological constraints while being indicated against small-cell lung cancer [40]. The quick conversion of the TPT bioactive lactone form to its inert carboxylate form was improved by the development of a PLGA nano-system encasing TPT, which enhanced its therapeutic efficacy [41]. The nano-system was tested, including cytotoxic effectiveness, and further TPT stability was maintained by preserving an acidic pH in the drug-containing phase in the nano-system. The drug was stabilized to stay in its lactone form and displayed a release pattern for 15 days due to the maintenance of a low pH inside the nanoparticles. Additionally, in vivo antitumor effects and in vitro cytotoxicity testing (using the LLC cell line) showed considerable potential for greater proliferation inhibition as contrasted with the pure drug TPT. In conclusion, the study identified a novel, straightforward method for creating stable TPT NPs that may be delivered effectively for lung cancer treatment.

Numerous cancer forms, including non-small-cell lung cancer, can develop epithelial-mesenchymal transition (EMT), which can promote metastasis [42,43]. Consequently, employing therapeutic nanoparticles to prevent EMT in lung cancer might be a novel approach. In one study, salinomycin (SAL) was delivered using polymeric micelles, which was demonstrated to inhibit EMT in lung cancer, resulting in a decrease in the ability of A459 lung cancer cells to migrate without impairing cell growth [44]. A459 lung cancer cells’ ability to spread through EMT was decreased by using silver nanoparticles as nanocarriers to carry therapeutic compounds such as gallic acid [45].

### 2.2. Active Targeting of Nano-Formulations for Lung Cancer

Active targeting promotes drug delivery effectiveness by increasing the cellular internalization of NPs at tumor locations [46,47]. The said approach is possible by surface decorating the NP to bind exactly to cell receptors on tumor cells. The effectiveness of the treatment is improved, adverse effects are reduced, the drug amount at the tumor location is increased, and the dosage of the drug to be administered is decreased [48]. A plethora of chemotherapeutics has been reported to be designed to deliver to tumor areas using various targeted nano-drug delivery systems by focusing on overexpressed receptors on the tumor surface.

For NSCLC-targeted drug administration, an RGD peptide-modified chitosan-based nanoparticle formulation (CSNP)-RGD was developed [49]. Chitosan was employed to boost adhesion, limit drug release, and enhance particle stability. The integrins αvβ3 and α5β1 on the surface of cells are recognized by the linear peptide known as GRGDSP. The targeted formulation entrapping paclitaxel was 217 nm in size. Since integrin αvβ3 overexpression is present in NSCLC cells such as A549 and H1299, PTX-PLGA-CSNP-RGD was very selective for these cells. The formulation entered cells through endocytosis, which was regulated by integrin αvβ3. This protein suppressed the G2/M cell cycle and triggered apoptosis in tumor cells, although it was noted to be mostly nontoxic to healthy bronchial epithelial cells. In vivo experiments showcased that GRGDSP inhibited tumor growth and minimized detrimental implications. According to this study, PLGA-CSNP-RGD is predicted to be a technique for delivering drugs to lung cancer cells in a prioritized manner.

To effectively treat lung cancer, a group of researchers in 2017 synthesized multi-wall carbon nanotube loaded with chitosan-folate-conjugated digitoxin. The cytotoxicity assay revealed substantial intracellular levels and enhanced cellular internalization of the nanocarrier. By incubating in A549 cells, the nano-formulation reached 89 times the therapeutic efficacy in IC50 measures compared with the commercial product DOCELTM. The targeted and untargeted formulations exhibited lower toxicity profiles and offered a promising framework for effective lung cancer therapy [50].

Doxorubicin (Dox) conjugated on the surface of gold nanoparticles with PVP was formulated. Compared to free doxorubicin, they found that the targeted formulation combination significantly increased cellular penetration with a notable intracellular release of Dox when evaluated in lung cancer cells [51].

To aid in the diagnosis, staging, prognosis, and therapeutic usage, quantum dots (QDs) may be crucial to the imaging of tumors [52]. QDs are promising prospects for constructing multimodal theranostics due to their distinct optical characteristics and ability to functionalize with biomolecules [53]. Multispectral investigations showed a narrow fluorescence emission (570 nm) following stimulation (400 nm). As a result, it may be stated that doxorubicin-conjugated bi-functionalized InP/ZnS QD may be employed as a theragnostic for lung cancer treatment and diagnostics at the same time [54].

Another NP that uses hyaluronic acid (HA) as a targeting agent is DTX/PPN@PPY@HA. The nanoparticle consisted of a fatty-acid phase-changing core and an exterior layer made of photoresponsive polypyrrole, HA, and docetaxel [55]. Laser activation of polypyrrole in tumor cells, along with irradiation exposure, induces local hyperthermia, which leads to the melting of the fatty acid core ending up in drug release that is activated by heat. The research shows that NP DTX/PPN@PPY@HA demonstrated outstanding photothermal chemotherapeutic efficacy with a superior cellular absorption profile. PPN@PPY@HA did not cause cytotoxicity in vitro or harm healthy tissue when evaluated in vivo. Additionally, in vivo evaluations showed that the intra-tumoral injection of NPs was capable of total tumor eradication, while intravenous administration suppressed tumor growth.

Table 1 includes references to a selection of the research that has been covered in Section 2.1 and Section 2.2.

## 3. Drug Resistance in Lung Cancer

**Figure 4:**
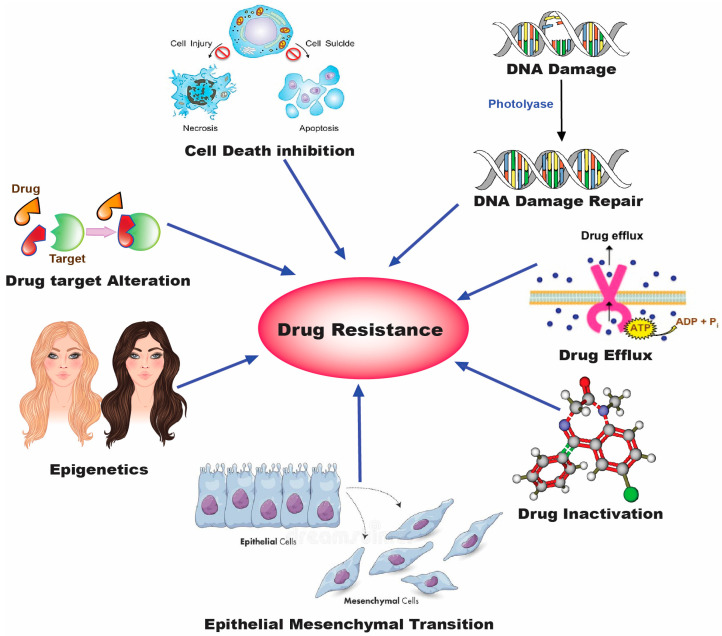
Various techniques employed by human cancer cells for direct or indirect drug resistance.

Due to the emergence of drug resistance in cancer cells, the therapeutic potential of chemotherapeutics is known to be constrained [56]. Drug resistance refers to the tumor cells’ capacity to generate a specific strategy to counteract and suppress chemotherapeutics’ cytotoxic or inhibitory action, lowering their therapeutic efficacy. Approximately 90% of clinical metastasis occurrences result from chemotherapeutic failure due to drug resistance. Chemotherapeutic drugs must be provided at larger doses and more frequently to combat resistance, which may lead to significant toxicity and a decrease in the overall patient survival rate. To establish a synergistic impact and lower the rate of resistance, two or more chemotherapeutics drugs may be combined to achieve potentially high therapeutic effectiveness [57]. The various factors responsible for the emergence of drug resistance in tumor cells are presented in Figure 4.

Conventional cancer therapy in LC remains confronted with significant challenges, including tumor multidrug resistance (MDR) [58]. The 48 known genes that make up the ATP-binding cassette (ABC) membrane pumps have been connected to several MDR processes [59]. BCRP and P-gp are a few of these efflux transporters that have been known to lessen the effectiveness of chemotherapeutics in tumor cells by notably curbing their intracellular accrual in an ATP-dependent fashion. Taxanes, platinum-based drugs, and gemcitabine, which are frequently used chemotherapeutic drugs, are susceptible to the said pathways [60].

The initiation, development, and poor prognosis of NSCLC have all been linked to the upregulation of EGFR signaling, which is brought on by gene mutations, gene amplification, or both [61]. EGFRC797S and EGFRG724S mutations, MET/HER2 amplification, activation of the RAS-mitogen-activated protein kinase (MAPK) or RAS-phosphatidylinositol 3-kinase (PI3K) pathways, new fusions, and histological transformation are a few examples of the numerous EGFR-dependent and EGFR-independent resistance mechanisms that have been discovered [62]. In lung cancer cells, the upregulation of EGFL7 also promotes NOTCH signaling, which slows the decline in c-Myc produced by EGFR inhibition and aids in cancer cell survival [63].

In patients with advanced non-small-cell lung cancer (NSCLC) with EGFR oncogene addiction, osimertinib is an irreversible, third-generation EGFR tyrosine kinase inhibitor that is extremely selective for EGFR-activating mutations as well as the EGFR T790M mutation [64]. Osimertinib has been shown to be effective in first- and second-line settings, but patients always acquire resistance, leaving them with no straightforward alternative therapeutic options other than chemotherapy and, in some cases, locally ablative therapy. The acquired osimertinib resistance is highly variable, covering EGFR-dependent as well as EGFR-independent mechanisms, as a result of the high degree of tumor heterogeneity and adaptive cellular signaling pathways in NSCLC.

Long non-coding RNAs (lncRNAs), a diverse class of regulatory molecules that regulate genes and signaling pathways associated with cell growth, metastasis, and medication response, have received a great deal of interest [65].

Patients with later episodes of LC are frequently treated with platinum-based compounds such as cisplatin. ROS and DNA damage is produced when the chemotherapeutics enter cancer cells, which results in cellular apoptosis [66]. By rendering the platinum medications ineffective through various resistance processes, including increased DNA repair, decreased cellular internalization, and anti-apoptosis, the resistance to LC inevitably evolves. According to several research investigations, the active SH-group in glutathione has an affinity to attach to platinum-based pharmaceuticals and prevent their DNA targeting owing to their strong interaction with anticancer medications. This can prevent DNA targeting. The cells become cisplatin-resistant because of the increased drug efflux through the GS-X pump and the increased glutathione content in the cells [67].

In both in vitro and in vivo research on LC, previous findings investigated the possible use of nano-systems in overcoming many MDR pathways. Because NPs can permeate cells in high numbers through endocytosis rather than diffusion, and release drugs inside the cell at a perinuclear region far from the efflux pumps, it has been hypothesized that chemotherapeutic-entrapped NPs can circumvent efflux transporters [68]. Another method entails delivering the chemotherapeutic drug and P-gp inhibitor via nano-enabled systems.

Through the encapsulation of genetic materials, the application of NPs to impede different MDR mechanisms in LC has been investigated [69]. Combinatorial therapy using survivin siRNA along with Pt (IV) prodrug delivered into a polyglutamic acid (PGA)-coated protamine/hyaluronic acid nanocarrier was investigated [70]. The combinatorial formulation conferred tumor suppression rates of approximately 82.46% when evaluated on nude mice bearing the A549/DDP tumor. However, the tumor regression rates in groups of mice treated with free cisplatin were noted to be 62.52% after 14 days of treatment. The results demonstrated the better efficacy reports of the combination drug therapy than the neat drug.

Albumin-based cationic NPs with hyaluronic acid functionalization loaded with all-trans-retinoic acid (ATRA) were developed and examined in vivo lung metastasized tumor models with CD44 overexpressed cancer stem cells. According to the biodistribution profile, the HA-decorated NPs were selectively taken up by the mouse tumor tissue and significantly slowed tumor development compared to the unmodified medication [71].

The overexpression of the ABC pumps in resistant LC cells following paclitaxel (PTX) treatment resulted in the proliferation of genes in the MDR1/ABCB1 chromosomal domain that code for P-gp. This led to PTX’s intracellular aggregation being reduced, its egress from cancer cells increasing, and the emergence of drug resistance [72]. Chemotherapeutic drugs can avoid the ABC drug efflux pumps by being encapsulated in nanoparticles or conjugated to polymeric nanocarriers, rendering them unidentifiable as export platforms. DOX was entrapped in liposomes containing anti-MRP-1 and anti-Bcl2 siRNA. The nanocarrier system reduced the activity of efflux pumps with a notable internalization of the chemotherapeutics in resistant LC cells [73].

## 4. Combinatorial Therapy for Lung Cancer Treatment

For resistant tumors, combination therapy using various chemotherapeutics is effective due to its synergistic action, reduced toxicity, and lack of drug resistance [74]. Other combination strategies also include photothermy, hyperthermia, or ultrasounds, in combination with stimuli-responsive nanoparticles [75,76]. Platinum drugs, which were frequently used with paclitaxel, docetaxel, gemcitabine, or irinotecan, in addition to radiation, continue to be the most effective treatment for advanced NSCLC [77]. However, the unique physicochemical characteristics and in vivo pharmacodynamics and pharmacokinetics of the various drugs present challenges for combinatorial therapy, making it difficult to optimize dose and administration timing [78]. Exploiting various nanotools in such a pursuit curtails the associated challenges and presents enhanced therapeutic effectiveness. Table 2 represents an array of combinatorial drug therapy approaches utilizing passive and active targeting approaches to combat lung cancer.

Research studies have been conducted on the ability of nanoparticles to transport the immune modulators that function in immunotherapy. In this regard, some studies have suggested that nanoparticles may transport molecules that successfully modulate the immune response. In such a pursuit, ARAC (Antigen Release Agent and Checkpoint Inhibitor), a nanoparticle-based immunotherapy created to boost the effectiveness of PD-L1 inhibitors, was developed [88]. ARAC is a nanoparticle that simultaneously delivers the PD-L1 antibody and PLK1 inhibitor, volasertib. In a metastatic lung tumor model (LLC-JSP), ARAC was noted to reduce the effective doses of volasertib and PD-L1 antibody by five times, and the impact was mostly mediated by CD8+ T cells. Another lung tumor model (KLN-205), which was unresponsive to the combination of CTLA-4 and PD-1 inhibitors, also demonstrated efficacy when ARAC was delivered. This research utilized the multi-cargo nanoparticle platform, which can load different cargo types simultaneously, to highlight a logical combination strategy to supplement already available therapies.

It has been suggested that celastrol-nanoparticle-containing M1-like macrophages (NP@M1) could be used as a combination therapy [89]. Celastrol was delivered by M1-like macrophages (M1), which also acted as a biotherapeutic agent. Additionally, it was shown that celastrol nanoparticles (NPs) maintained the anticancer-polarized condition of M1 and that the exocytosed NPs also carried out the tumor cell-killing function.

A report on the production of magnetic nanoparticles with maghemite as its core and three distinct coatings, namely dextran, carboxymethyl dextran, and dimercaptosuccinic acid, was published [90]. It has been demonstrated that CMD-MNP-miRNA155 and CMD-MNP-miRNA125b nanoparticles may cause human macrophages to express more CD80 and have higher levels of TNF- and IL-6. As a result, the suggested magnetic nanoparticle-based miRNA-delivery nanosystem can be used as a new immunotherapeutic tool.

## 5. Clinical Studies of Nanocarriers in Lung Cancer

Progressive forms of LC typically necessitate a standard combination of chemotherapy and radiotherapy treatment in conjunction with additional cutting-edge treatments such as immunotherapy or tailored therapeutics. Nevertheless, the usage of these agents is complicated by resistance tendencies and combination perspectives. We currently have a huge number of clinical trials under development that attempt to bring the developments in NPs for these cancers to the clinical phase (Table 3).

In the trial BIND-014, total docetaxel (DTXL) plasma concentrations remained at least 100-fold greater than solvent-based docetaxel (sb-DTXL) for more than 24 h in tumor-bearing mice, rats, and nonhuman primates treated with targeted docetaxel (DTXL-TNP) [91]. These results were consistent with an extended circulation of NPs in the vascular compartment and regulated release of DTXL. DTXL-TNP exhibited a pharmacological profile distinct from sb-DTXL, including pharmacokinetic character traits consistent with preclinical data and instances of tumor shrinkage at doses below the dose of sb-DTXL usually used in the clinic, according to preliminary clinical data in patients with advanced solid tumors.

For clinical study NCT00077246, on days 1, 8, and 15 of a 28-day cycle, NAB-paclitaxel at a dose of 125 mg/m^2^ was given, and it showed encouraging single-agent activity [92]. No premedication with corticosteroids was given, and no hypersensitive reactions were seen. It was needed to conduct more research on both platinum-based combinations and single-agent NAB-paclitaxel.

## 6. Challenges Associated with the Use of Nanomedicine in Cancer

Even though the field of nanomedicine has been revolutionized in the past decade, some appended caveats in the domain hinder the widespread usage and the clinical applicability of nano-based therapies. Passive and active targeting are the two approaches utilized for accumulating chemotherapeutics at the tumor zone. However, certain challenges need to be defined ahead of their clinical applicability.

### 6.1. Challenges Associated with Non-Targeted (Passively Targeted) Nano-Formulations

First-generation passively tailored nanomedicines make up most nanocarrier-based cancer treatments [93]. The pathophysiological characteristics of cancers and the environment around them have been employed for passive targeting, especially in cases when the EPR effect substantially encourages the buildup of nanomedicine in cancer cells. As a result, the passive targeting of neoplasms using nanomedicine is possible through diffusion and convection without the need to attach a specific chemical to the surface of the nanocarrier. However, it is generally acknowledged that directed delivery offers more advantages than passive targeting based on EPR effects on cytotoxic medication side effects.

The delivery of drugs through a passive targeting approach may be significantly impacted by the complexity of cancer and its stroma, such as hypoxic gradients, leading to decreased or eliminated transport of substances into neoplasms [94]. Recent studies have concentrated on standardizing the neoplasm vasculature before beginning cancer treatment. Furthermore, passive targeting does not stop the aggregation of nanocarriers in former fenestrated endothelial organs such as the liver and spleen, supporting next-generation nanomedicine development with cutting-edge practicalities [95].

### 6.2. Challenges Associated with Targeted (Ligand Anchored/Active Targeted) Nano-Formulations

Actively targeted nano-formulations used to deliver macromolecules encounter extra physiological obstacles due to their interactions with the target cells. Escape from the endocytic route is one of the main obstacles noted. Intracellular trafficking pathways drive NCs to subcellular regions following endocytosis, which may harm the fate of nano-formulations. For instance, transferrin-targeted nano-formulations that are ingested through clathrin-mediated endocytosis would transit the degradative route and ultimately be digested in lysosomes [96]. Various methods have been explored, including pore-formation peptides, proteins, and pH-buffering compounds that use the “proton sponge effect” to help nano-formulations egress from endosomes and enter the cytosol. The endosomal escape of NCs in vivo is still exceptionally challenging, though.

The enormous diversity within and between tumors and the prevalence of tumor and metastasis-supportive stroma add to the complexity [97]. Many active, cell-specific nano-formulations ignore tumor heterogeneity and favor the persistence of resilient clones by targeting a single cell-surface receptor on cancer cells. Because of this, current therapy frequently produces visible partial or complete cures, which are typically followed by resistant tumor recurrence and mortality [98].

Approaches for active targeting are substantially more intricate than those for passive methods. To understand the challenges brought on by physiological barriers and tumor heterogeneousness, a significant barrier is presented by the intricate design and manufacturing of these nano-formulations, which may make the scale-up steps more difficult [99]. Due to the significant processes of chemical synthesis and purification, the manufacturing process for conjugating ligands targeting nanocarriers is more complicated than it is for passively targeted ones.

Despite not being the main obstacle to the clinical translation of actively targeted nanocarriers, this does provide a substantial difficulty for the bench-to-bed translation of this strategy, because it requires more quality control processes, is more expensive, and takes longer to complete. For instance, a lipidated single-chain Fv-based linker technology that can self-assemble into lipid-based nanocarriers and bind IgGs of a specific isotype was formulated by a group of researchers [100]. The intricacy of the chemical conjugation of antibodies to nanocarriers and the requirement for the removal of unbound antibodies were both resolved by the integration of the linker to the lipidic system via self-assembly. A single adaptable nanocarrier platform that can be directed to several cellular targets by combining with various targeting moieties may also be created by using linkers that can bind various targeting moieties. Without complicating the process of producing nanocarriers, such solutions might offer a real advance in overcoming the problems caused by tumor heterogeneity.

Immunological and hematological problems are significant issues with cancer nanomedicine and its advancement. Metallic nanoparticle translation is initially concerned with specific responses known as anaphylactic reactions [101]. These issues are connected to the polymeric coating materials employed to eliminate the agents present in their commercialized forms. Furthermore, endotoxin contamination and related complications such as activation and pyrexia are crucial problems when employing nanomedicine as therapeutics and cancer therapies.

The toxicity aspects of nanoparticles are an important factor to consider as they impede clinical translation [102]. Electrostatic interactions between nanoparticles with positive surface charges and pulmonary surfactants have been demonstrated [103]. It has been hypothesized that these interactions may alter how nanoparticles interact with cells. The lack of vesicles encircling the ingested nanoparticles or nanoparticle aggregates revealed that the nanoparticles entered the cells via a non-endocytosis pathway. The membrane was also harmed at the point where the cells entered [104]. In such cases, suitable surfactants need to be employed during the formulation of nanoparticles which may help in reducing the toxicity impacts.

Animal models must be used in order to evaluate in vivo NP performance, such as PK, biodistribution, effectiveness, and safety. Although some research findings have shown PK scaling for various nanotherapeutics across different species (including humans), one well-recognized barrier is the significant disparity between the efficacy of treatment obtained in preclinical models and the results from clinical trials [105]. This is largely because there are not enough tumor models that can accurately mimic human cancers. Various animal models are currently available, including patient-derived xenografts (PDXs), genetically modified mice, and cell-line-based subcutaneous and orthotopic xenografts (GEMMs). EPR is typically more consistent in animal models than in cancer patients, but no one model can completely replicate all features of human malignancy [106]. Additionally, models of human tumor metastasis will be crucial for assessing EPR and NP permeation and targeting in metastatic tissues compared to primary tumors, given the significant role that tumor metastases play in cancer mortality. The establishment of animal models that substantially resemble the heterogeneity and anatomical histology of human malignancies may greatly enhance the translation of nanotherapeutics.

## 7. Recent Patent Literature: Innovative Approaches and Novel Formulations

The patent search was performed on freely available patent databases such as Espacenet, Patent Scope, and USPTO [107,108,109]. This patent search revealed many patents/patent applications related to using nanoparticles of anticancer agents to treat lung cancer. This patent search aimed to highlight some nanoparticle-based inventions for treating lung cancer. A few patented nanoparticle-based lung treatments are summarized in Table 4.

## 8. Conclusions

Even though several NPs have made significant strides toward becoming possible treatments, most of them still fall short of the required clinical requirements. Additionally, clinically authorized NPs have demonstrated efficacy in lowering drug toxicity; nevertheless, their usage has not always led to superior clinical outcomes. The clinical translation of nanomedicines has been threatened by a lack of comprehension of biochemical pathways, complex structure, a lack of precise characterization methods, as well as the high cost of manufacture. Hence, many of them are still in pre-clinical phases because it is challenging to construct nano-formulations with various functionalities and a uniform size distribution for enhanced performance.

The therapeutic prospect of passive targeting is relatively substantial, but it is currently limited by the physiologic challenges associated with the EPR effect’s unpredictability. To ascertain the EPR effect, efficient quantitative EPR imaging equipment must be developed. It is imperative to explore the application of these imaging modalities on patients since it may be a practical way to gather vital information for the engineering of the next nano-formulations. These tools may also provide practitioners with additional diagnostic tools that will aid in the early identification of patients who might benefit from EPR-based therapeutics, hence maximizing the efficacy of their interventions. The intricate design and cellular barriers to proper intracellular delivery of the entrapped drug are additional challenges specific to the actively targeted nano-formulation that limit their therapeutic usefulness.

The accretion of a protein corona in biological conditions and its effects on pharmacokinetics and pharmacodynamics should also be considered when designing passively or actively targeted nano-formulations. It takes innovative methods to develop nano-formulations with tunable biological identities in order to accelerate clinical translation. Other design issues, such as the responsiveness of the nano-formulations to the stimuli, scale-up, and associated toxicity of the nano-formulations, should also be meticulously considered when developing stimuli-responsive nano-systems to minimize untimely release.

Furthermore, it is crucial to research nano-formulations’ in vivo toxicity and biodistribution before taking them up to clinical studies. With the improved comprehension of tumor biology, and the discovery of genuine biomarkers that can foretell responders and non-responders, the overall effectiveness of introducing innovative nano-formulations into clinical settings is likely to increase.

Finally, the recent patent literature gives hope as many new approaches and formulations are being patented. Researchers are working hard to develop new combinatorial drug delivery systems that use nanotechnology to help treat lung cancer.

## Figures and Tables

**Table 1:** The table summarizes the list of studies discussed in Section 2.1 and Section 2.2.

Nanocarrier	Drug	Targeting Ligand	Result	Ref.
Polymeric nanoparticles	Paclitaxel; transcription-3 (Stat-3) siRNA	—	A combinatorial formulation inhibited Stat-3 expression and increased cellular death by accumulating paclitaxel in A549 cells.	[39]
Polymeric nanoparticles	Topotecan	—	The drug was stabilized to stay in its lactone form and displayed a release pattern for 15 days due to the maintenance of a low pH inside the nanoparticles. Additionally, in vivo antitumor effects and in vitro cytotoxicity testing (using the LLC cell line) showed considerable potential for greater proliferation inhibition as contrasted with the pure drug TPT.	[41]
Micelle	Salinomycin	—	Inhibited EMT in lung cancer, resulting in a decrease in the ability of A459 lung cancer cells to migrate without impairing cell growth.	[44]
Silver nanoparticles	Gallic acid	—	A459 lung cancer cells’ ability to spread through EMT was decreased.	[45]
Polymeric nanoparticles	Paclitaxel	RGD peptide	In vivo experiments showcased that GRGDSP inhibited tumor growth and minimized detrimental implications.	[49]
Carbon nanotube	Digitoxin	Folate	The cytotoxicity assay revealed substantial intracellular levels and enhanced cellular internalization of the nanocarrier. By incubation in A549 cells, the nano-formulation reached 89 times the therapeutic efficacy in IC_50_ measures when compared with the commercial product DOCELTM.	[50]

**Table 2:** List of combinatorial drug therapy approaches utilizing the passive and active targeting strategy to combat lung cancer.

Drug	Nanocarrier System	Targeting Ligand	Tumor Model	Therapeutic Effectiveness	Ref.
Gefitinib and vorinostat	Polymeric nanoparticles	Hyaluronic acid	Female NMRI nude mice	Targeted delivery to subcutaneous CD44-overexpressing tumors. Co-delivery of the drugs employing the nanoparticles decreased the cytotoxic effects of the native drugs. Compared to free drugs, intrapulmonary delivery of dual drug-loaded nanoparticles exhibited a better orthotopic lung tumor growth reduction.	[79]
Doxorubicin and cisplatin combined with siRNA	Mesoporous silica nanoparticles	LHRH peptide	NCR nude mice	By limiting their buildup in other non-target organs and preventing their egress into the systemic circulation, the local biodistribution of the formulation by inhalation contributed to the predominant deposition of NPs at the target site.	[80]
Paclitaxel + Cisplatin	Micelle	—		When exposed to an acidic reducing environment (pH 5.5 + dithiothreitol), about 100% of both medications were generated within 192 h. Compared to the free drugs, the dual drug-loaded micelles caused 1.77 times more cellular killing when evaluated in NCI-H520 LC cells.	[81]
NU7441 - a potent radiosensitizer and gemcitabine	Polymeric nanoparticles	—	H460 tumor-bearing mice	Biphasic release of NU7441, as well as pH-dependent gemcitabine release, was noted. Superior hemocompatibility, along with remarkable dose-dependent caveolae-mediated in vitro internalization, was observed in lung cancer cells.	[82]
Paclitaxel and cisplatin	Lipid-polymer nanoparticles	RGD peptide	lung tumor xenografts (A549 cells injected into female BALB/c mice).	IC_50_ values of 26.7 and 75.3 g/mL for dual drug-loaded nanoformulation and free drugs, respectively, indicated much stronger anticancer activity. Regression of tumor size from 1486 mm^3^ to 263 mm^3^.	[83]
Docetaxel prodrug (DTXp) and cisplatin (DDP)	Lipid–polymer hybrid nanoparticles	Aptamer	lung tumor xenografts (A549 cells injected into female BALB/c mice).	Comparing APT-DTXp/DDP-LPHNs to non-aptamer-functionalized LPHNs and single drug-entrapped LPHNs, these LPHNs demonstrated significantly improved cytotoxicity with synergistic antitumor impact with a combination index of 0.62 and substantial tumor-inhibition capability.	[84]
Paclitaxel (PTX) and triptolide (TL)	Lipid–polymer hybrid nanoparticles (LPNs)	—	lung tumor xenografts (A549 cells injected into female BALB/c mice).	When the PTX: TL weight ratio was 5:3, the combinatorial therapy showcased synergistic benefits. The experimental group’s in vivo tumor development curve was less pronounced than that of the control group, and the tumor volumes in the P/T-LPNs and control groups were observed to be 392 and 1737 mm^3^, respectively.	[85]
Doxorubicin and cis-platinum	Polymeric nanoparticles	—	B16F10 tumor-bearing mouse models	The co-delivery system showed more cytotoxic potential than patients treated with either of the neat drugs alone, according to in vitro cytotoxicity studies performed on the B16F10 cell line. Local delivery of combinatorial drug therapy by pulmonary injection in B16F10 tumor-bearing mouse models showed that dual drug-loaded NPs had highly effective deposition in the lungs but infrequently in normal lung tissues.	[86]
Epirubicin and paclitaxel	Polymeric nanoparticles	—	—	Compared to the native drugs, the MTT assessment revealed that PLGA-PEI-EPI-PTX NPs had a substantial amount of antitumor effects. Additionally, a Western blot analysis of the expression of the p53 protein revealed increased expression.	[87]

**Table 3:** List of clinical studies indicating the usage of chemotherapeutic-entrapped nanocarriers for lung cancer treatment.

Intervention	Nanocarrier	Trial Phase	Identifier No.	Primary Endpoint	Status
BIND-014	Prostate-specific membrane antigen targeted nanoparticles	II	NCT01792479	Number of patients with either a complete or partial response	Completed
Drug: HLX10 Drug: carboplatin and nab-paclitaxel Drug: Placebo	Nanoparticle albumin bound (Nab) Paclitaxel	III	NCT04033354	Tumor assessment Progression-free survival (PFS)	Active, not recruiting
Drug: carboplatin Drug: erlotinib hydrochloride Drug: paclitaxel albumin-stabilized nanoparticle formulation Radiation: radiation therapy	Paclitaxel albumin-stabilized nanoparticle	II	NCT00553462	Determination of therapeutic activity	Completed
Drug: CRLX101 Other: Best Supportive Care	Camptothecin-conjugated polymeric nanoparticle	II	NCT01380769	To compare the overall survival of patients treated with CRLX101 + BSC to those patients treated with BSC only Comparison of survival among patients treated with CRLX101 + best supportive care vs. patients treated with best supportive care only	Completed
Drug: Paclitaxel (Genexol^®^) Drug: Paclitaxel loaded polymeric micelle (Genexol-PM^®^)C	Micelle	II	NCT01023347	The response rate to therapy	Completed
Drug: LY01610 (Irinotecan hydrochloride liposome injection)	Liposome	II	NCT04381910	Objective response rate (ORR) Duration of response (DoR)	Recruiting
Drug: Irinotecan liposome injection Drug: Topotecan	Liposome	II/III	NCT03088813	Overall survival (OS)	Active; not yet recruiting
Drug: paclitaxel albumin-stabilized nanoparticle formulation	Albumin stabilized nanoparticles	I/II	NCT00077246	Maximum-tolerated dose (MTD) and dose-limiting toxicity (DLT) of ABI-007 Objective target lesion response	Completed
Biological: quaratusugene ozeplasmid Drug: osimertinib	Lipid nanoparticles	I/II	NCT04486833	Recommended phase 2 dose (RP2D)	Recruiting

**Table 4:** The summary of nano-particle-based patents/patent applications for lung cancer.

Patent/Application Number (Applicant)	Type of Nanocarriers	Drugs	Targets	Ref.
**US8912212B2** (Bind Therapeutics)	Polymeric nanoparticle	Docetaxel	Non-Small-Cell Lung Cancer	[110]
**US8236330B2** (Bind Biosciences)	Polymeric nanoparticle	Docetaxel	Non-Small-Cell Lung Cancer	[111]
**US8603500B2** (Bind Therapeutics)	Polymeric nanoparticle	Paclitaxel/Docetaxel/Doxorubicin/gemcitabine/5-Fluorouracil/Daunorubicin/9-Dihydrotaxol	Non-Small-Cell Lung Cancer	[112]
**US9393318B2** (Abraxis Bioscience)	Paclitaxel albumin-bound nanoparticles	Paclitaxel/Carboplatin	Non-Small-Cell Lung Cancer	[113]
**US11318131B2** (Ipsen Biopharm)	Irinotecan-Loaded Liposome	Irinotecan	Non-Small-Cell Lung Cancer	[114]
**US9895365B2** (Ipsen Biopharm)	Parenteral Liposomal Irinotecan	Irinotecan with other chemotherapeutics, e.g., Poly(ADP-ribose) Polymerase (PARP) inhibitor (niraparib, olaparib, veliparib, rucaparib, and talazoparib	Small-Cell Lung Cancer and Non-Small-Cell Lung Cancer	[115]
**CN104524565B** (Nanhua University)	PLGA-based polymeric nanoparticles	Dermatophagoides pteronyssinus-1 (Der p1) albumen	Epithelial Barrier of Lung	[116]
**CN105640986A** (Guangdong University of Technology)	Silver nanoparticles	Nano-silver and pharmaceutically acceptable Injection excipients (a suspending aid, a dispersing agent, a surfactant, an analgesic, a pH adjustment buffer agent, an osmotic pressure regulator, and water).	H1299 cells	[117]
**CN114557979A** (Medium-Mountain University)	Hesperidin-Lecithin Liposome	Hesperidin	A549 lung cancer cells	[118]
**CN105726483A** (Lu Kaihua)	Sunitinib and Smallanthus sonchifolius diterpene acid-Loaded Liposome	Sunitinib and Smallanthus sonchifolius diterpene acid	Pleural Mesothelioma	[119]
**CN103393598A** (Nanjing University of Chinese Medicine)	Triptolide liposome	Triptolide	Non-Small-Cell Lung Cancer	[120]

## Data Availability

The data for this article was collected from the references cited in the manuscript.
